# The minimal clinically important difference of app‐based electronic patient‐reported outcomes for hay fever

**DOI:** 10.1002/clt2.12244

**Published:** 2023-05-01

**Authors:** Ken Nagino, Jaemyoung Sung, Akie Midorikawa‐Inomata, Atsuko Eguchi, Keiichi Fujimoto, Yuichi Okumura, Alan Yee, Kenta Fujio, Yasutsugu Akasaki, Tianxiang Huang, Maria Miura, Shokirova Hurramhon, Kunihiko Hirosawa, Mizu Ohno, Yuki Morooka, Hiroyuki Kobayashi, Takenori Inomata

**Affiliations:** ^1^ Department of Hospital Administration Juntendo University Graduate School of Medicine Tokyo Japan; ^2^ Department of Ophthalmology Juntendo University Graduate School of Medicine Tokyo Japan; ^3^ Department of Digital Medicine Juntendo University Graduate School of Medicine Tokyo Japan; ^4^ University of South Florida Morsani College of Medicine Tampa Florida USA; ^5^ Juntendo University Graduate School of Medicine AI Incubation Farm Tokyo Japan

**Keywords:** hay fever, minimal clinically important difference, mobile health, patient‐reported outcome measures, pollen allergy

## Abstract

**Background:**

Hay fever is a common allergic disease, with an estimated worldwide prevalence of 14.4% and a variety of symptoms. This study assessed the minimal clinically important difference (MCID) of nasal symptom score (NSS), non‐nasal symptom score (NNSS), and total symptoms score (TSS) for app‐based hay‐fever monitoring.

**Methods:**

MCIDs were calculated based on the data from a previous large‐scale, crowdsourced, cross‐sectional study using AllerSearch, an in‐house smartphone application. MCIDs were determined with anchor‐based and distribution‐based methods. The face scale score of the Japanese Allergic Conjunctival Disease Standard Quality of Life Questionnaire Domain III and the daily stress level due to hay fever were used as anchors for determining MCIDs. The MCID estimates were summarized as MCID ranges.

**Results:**

A total of 7590 participants were included in the analysis (mean age: 35.3 years, 57.1% women). The anchor‐based method produced a range of MCID values (median, interquartile range) for NSS (2.0, 1.5–2.1), NNSS (1.0, 0.9–1.2), and TSS (2.9, 2.4–3.3). The distribution‐based method produced two MCIDs (based on half a standard deviation, based on a standard error of measurement) for NSS (2.0, 1.8), NNSS (1.3, 1.2), and TSS (3.0, 2.3). The final suggested MCID ranges for NSS, NNSS, and TSS were 1.8–2.1, 1.2–1.3, and 2.4–3.3, respectively.

**Conclusions:**

MCID ranges for app‐based hay‐fever symptom assessment were obtained from the data collected through a smartphone application, AllerSearch. These estimates may be useful for monitoring the subjective symptoms of Japanese patients with hay fever through mobile platforms.

## 1 INTRODUCTION

Hay fever is one of the most common allergic diseases affecting approximately 30 million people in Japan alone.^1^ The global prevalence of hay fever is estimated at 14.4%, and this number is expected to increase in the future.^2^, ^3^ Hay fever presents with a wide variety of symptoms including allergic rhinitis, conjunctivitis, and dermatitis.^4^ It is also a highly multifactorial disease, influenced by three major categories of factors: (1) environmental, such as pollen count and particulate matter; (2) lifestyle‐related, such as diet, tobacco use, and exercise; and (3) host‐related, such as age and biological sex.^5^, ^6^, ^7^, ^8^ Currently, the main treatment for hay fever revolves around symptom suppression and progression through the long‐term use of antihistamines, steroids, and eye drops.^4^, ^9^ However, due to the variability in its pathological underpinnings, severity, and symptomologies, every patient responds differently to prescribed treatment. To provide an effective regimen, providers must closely monitor patients' response to treatment and preventive measures, as well as the aforementioned factors to tailor treatment choices.^10^

Assessment of interventional responses mainly comprises evaluation of patients' subjective symptoms and clinician's objective findings.^11^ For a highly variable disease such as hay fever, an approach to comprehensively quantify patients' subjective symptoms using patient‐reported outcomes (PROs) has been frequently utilized.^12^, ^13^ PROs allow providers to quantify one's health status related to a disease of interest through questionnaires with information on symptomology, treatment, and overall health provided directly by patients.^14^ For hay fever, commonly used PROs include the nasal symptom score (NSS), non‐nasal symptom score (NNSS), and their sum, referred to as the total symptom score (TSS).^15^ Additionally, several disease‐specific PROs related to patients' quality of life (QoL), including the Rhinoconjunctivitis Quality of Life Questionnaire (RQLQ) and the Japanese Allergic Conjunctival Disease QOL Questionnaire (JACQLQ), may further aid in the assessment of a patient's hay‐fever status.^13^, ^16^, ^17^ A holistic view on PROs may be particularly useful as a quantifiable metric that reflects a variety of hay‐fever manifestations, including ocular and nasal symptoms, as well as their resultant effects on QoL.^18^, ^19^ Longitudinal monitoring of PROs to evaluate changes may allow clinicians to objectively measure treatment efficacy for hay fever in a personalized manner.^13^, ^20^

In clinical trials, a novel treatment is generally accepted as more effective when the analysis of its targeted effect shows statistical significance compared to an existing alternative or control.^13^ However, statistical significance often does not equate to clinical significance, particularly for subjective clinical markers including PROs.^13^ In bridging the gap between statistical significance and clinical significance, researchers deploy the concept of minimal clinically important difference (MCID).^21^, ^22^ MCID represents the minimal improvement gap that can be regularly perceived as a benefit of value to patients, and an increase in PRO above the designated MCID would represent a clinically meaningful treatment effect.^21^, ^23^ The designated MCID values can vary between patient populations due to the differences in demographics, disease severity, and patient expectation.^24^, ^25^ Therefore, the determination of appropriate MCID values requires the selection of MCIDs calculated from data from a patient population as close as possible to the target patient population.^24^

Our previous studies have elucidated numerous aggravating factors and stratified the symptoms of hay fever using a mobile health (mHealth) smartphone app for hay‐fever research—AllerSearch—released in February 2018.^4^, ^12^, ^20^, ^26^ AllerSearch is equipped with a function to assess users' hay‐fever symptoms through collecting ePROs (electronic PROs), including the NSS and NNSS.^4^, ^12^, ^20^, ^26^ mHealth, to date, has shown great promise in monitoring daily symptoms and treatment effectiveness with minimal intrusion.^27^, ^28^, ^29^, ^30^, ^31^, ^32^, ^33^ Utilizing mHealth and a PRO with a widely agreed‐upon MCID value, it may be possible to accurately track meaningful changes in patients' symptoms and provide tailored treatments that are effective for individuals.^4^ However, robust MCID values for hay‐fever outcomes collected through AllerSearch are yet to be determined.

In this study, appropriate MCID values were determined for NSS, NNSS, and TSS for evaluation of the subjective symptoms of hay fever based on data collected through a mHealth smartphone app, AllerSearch.

## 2 METHODS

### 2.1 AllerSearch smartphone application

The self‐developed smartphone application, AllerSearch, was initially released as an iOS‐based application on ResearchKit in February 2018, and the Android version was released on August 26, 2020, under a consignment contract with Juntendo University Graduate School of Medicine and InnoJin, Inc., Tokyo, Japan.^4^, ^20^, ^34^ The AllerSearch is freely available on the Apple App Store and on Google Play.

### 2.2 Study design

MCIDs were calculated using both anchor‐ and distribution‐based approaches using data from a previous large‐scale, crowdsourced, cross‐sectional observational study conducted between February 1, 2018, and May 1, 2020 using AllerSearch.^4^ This study was approved by the Independent Ethics Committee of Juntendo University Faculty of Medicine (approval number: M17‐0061‐M02) and was conducted in accordance with the ethical standards laid down in an appropriate version of the Declaration of Helsinki (as revised in Brazil, 2013). All users provided informed consent electronically after being informed about the nature of the study and possible consequences.^4^

### 2.3 Data source

Data provided by participants were collected through the AllerSearch app (Supplementary Figure 1a) for this study.^4^ Previous studies have described the workflow of user‐data collection.^4^, ^20^, ^35^ Briefly, participants downloaded AllerSearch using their own App Store credentials. Among those who provided electronic consent, participants provided basic information, including that on demographics, medical history, lifestyle, hay‐fever status, and preventive behavior for hay fever. Subsequently, participants used the daily‐tasks function to receive daily assessments of the conjunctiva and to answer the questionnaire on hay fever including the NSS (Supplementary Figure 1b), NNSS (Supplementary Figure 1c),^15^ daily subjective symptoms including the level of stress due to hay fever, and the response (non‐hay fever, hay fever, and unknown) to the question “Do you have hay fever?”^4^ (Supplementary Table 1 and 2), JACQLQ,^17^ and work productivity. This study used data on answers to the questionnaires related to symptoms of hay fever that were entered for the first time in the app.

The NSS consists of five items pertaining to rhinorrhea, nasal congestion, nasal itching, sneezing, and interference with daily life.^26^ The NNSS consists of four items pertaining to itchy eyes, watery eyes, eye redness, and itchy ears and mouth.^26^ Each item of the NSS and NNSS is scored on a 4‐point scale as follows: 0 = no symptoms, 1 = mild symptoms, 2 = moderate symptoms, and 3 = severe symptoms.^15^ The TSS (0–27) was derived from the sum of NSS (0–15) and NNSS (0–12). The face scale score was scored on a 5‐point scale depicting emotions ranging from 0 (fine) to 4 (crying).^11^, ^17^ The stress level was scored on an 11‐point visual analog scale from 0 (none) to 10 (most stressful).

### 2.4 Inclusion and exclusion criteria

Participants who resided in Japan with reported hay fever were included in this study. Participants without hay fever, with an unknown diagnosis, with missing questionnaire data related to symptoms of hay fever, face scale, and stress level were excluded from this study.

### 2.5 Anchor‐based analysis

Anchor‐based methods are used an external indicator as an anchor to assign patients to clinically relevant categories.^23^, ^36^ An estimation of MCID for specific PRO measures is recommended to be based on multiple anchors.^37^ Here, the face‐scale score of JACQLQ Domain III and today's stress level owing to hay fever were used as anchors in determining MCIDs of NSS, NNSS, and TSS.

The face scale score was used as a 5‐scale anchor to categorize participants into five severity categories.^11^ There is no established method for determining meaningful change on an 11‐point stress level scale. In a prior study, a 2‐point change was used as a meaningful difference on an 11‐point scale.^23^, ^38^, ^39^ Based on this method, the level of stress was assessed according to a 5‐scale anchor and the participants were categorized into five severity groups.^39^ To assess anchor eligibility for determining MCID, Spearman's correlation coefficient and the number of participants in each severity category were calculated.^23^, ^39^ An anchor was considered suitable when the correlation coefficient between the anchor and total of NSS, NNSS, and TSS was ≥0.3 and the sample size within a severity category was at least 10 entries.^23^, ^39^

Mean differences between adjacent categories of the anchor provided estimates of the MCID. The anchor‐based MCIDs were estimated using medians with interquartile ranges (IQR).^23^, ^39^

### 2.6 Distribution‐based analysis

Distribution‐based methods were presented as supporting data to facilitate the interpretation of the anchor‐based results. The following two distribution‐based methods were used as an additional confirmation of the MCID.^37^

### 2.7 Standard error of measurement

The formula used for calculating the standard error of measurement (SEM) was “standard deviation (SD)*√(1 − r)”, where *r* was the recommended PRO test‐retest reliability.^23^, ^24^ In this study, intraclass correlation coefficient (ICC) was used as a metric for test‐retest reliability to determine SEM for calculation of MCID.^24^

ICCs were calculated by comparing baseline and subsequent measurements of NSS, NNSS, and TSS from patients who indicated no change in the face scale score and stress level.^40^ A PRO score difference smaller than the SEM is likely to represent an error of measurement rather than a real change.^23^, ^37^ In cases where the median anchor‐based MCID was less than SEM, the SEM was used as the MCID.^23^

### 2.8 Half a standard deviation

Half a standard deviation (SD) at baseline was assumed to be equal to the MCID.^41^, ^42^ The 0.5 SDs into each category were calculated. The calculated 0.5‐SD was used to determine MCID ranges.^43^

### 2.9 Statistical analysis

Ranges of MCID were recommended rather than single MCID estimate values.^23^ As such, the results of the anchor‐based MCID estimates calculated using medians with IQR and distribution‐based estimates based on 0.5‐SD and SEM were summarized as MCID ranges.^23^ To compare the characteristics of the participants in each group, continuous variables were presented as means, and SD and categorical variables were presented with proportions. The STATA software package (v. 17.0, Stata Corp, College Station, TX, USA) was used for all the analyses.

## 3 RESULTS

### 3.1 Participants' characteristics

In total, 7590 participants were analyzed in Tables 1 and 2. Supplementary Figure 2 shows the flow diagram for participant enrollment in this study. The mean age of the participants was 35.3 years (SD ± 13.9) and 57.1% (*n* = 4331) of the participants were women. Both the face score and stress level scale showed that the more severe the category, the higher the proportion of younger and female participants. Participants in the more severe group were more likely to state their medical history as unknown.

**TABLE 1:** Demographics and characteristics of participants by face scale score category.

	Face score 0	Face score 1	Face score 2	Face score 3	Face score 4	Overall
	*N* (%) = 667 (8.8)	*N* (%) = 1864 (24.6)	*N* (%) = 2755 (36.3)	*N* (%) = 1849 (24.4)	*N* (%) = 455 (6.0)	*N* (%) = 7590 (100)
Demographic characteristics						
Age, years, mean (SD)	37.7 (13.8)	37.6 (14.4)	35.4 (13.8)	33.1 (13.2)	30.3 (12.2)	35.3 (13.9)
Women, *N* (%)	265 (39.7)	1001 (53.7)	1610 (58.4)	1141 (61.7)	314 (69.0)	4331 (57.1)
Height, cm, mean (SD)	166.1 (9.0)	164.4 (8.7)	163.5 (8.8)	163.1 (8.8)	162.0 (8.8)	163.8 (8.8)
Weight, kg, mean (SD)	62.5 (12.3)	60.8 (12.2)	59.8 (12.2)	59.7 (12.4)	58.4 (12.2)	60.2 (12.3)
Medical history						
Medicated hypertension, *N* (%)						
No	551 (82.6)	1567 (84.1)	2263 (82.1)	1485 (80.3)	359 (78.9)	6225 (82.0)
Medicated	41 (6.2)	122 (6.6)	130 (4.7)	80 (4.3)	13 (2.9)	386 (5.1)
Unmedicated	33 (5.0)	60 (3.2)	91 (3.3)	61 (3.3)	13 (2.9)	258 (3.4)
Unknown	42 (6.3)	115 (6.2)	271 (9.8)	223 (12.1)	70 (15.4)	721 (9.5)
Diabetes, *N* (%)						
No	619 (92.8)	1707 (91.6)	2459 (89.3)	1644 (88.9)	390 (85.7)	6819 (89.9)
Yes	17 (2.6)	41 (2.2)	62 (2.3)	28 (1.5)	10 (2.2)	158 (2.1)
Unknown	31 (4.7)	116 (6.2)	234 (8.5)	177 (9.6)	55 (12.1)	613 (8.1)
Systemic diseases, yes, *N* (%)						
Blood disease	13 (2.0)	32 (1.7)	39 (1.4)	27 (1.5)	8 (1.8)	119 (1.6)
Brain disease	6 (0.9)	15 (0.8)	35 (1.3)	20 (1.1)	4 (0.9)	80 (1.1)
Collagen disease	2 (0.3)	8 (0.4)	19 (0.7)	8 (0.4)	4 (0.9)	41 (0.5)
Heart disease	18 (2.7)	38 (2.0)	53 (1.9)	37 (2.0)	9 (2.0)	155 (2.0)
Kidney disease	10 (1.5)	33 (1.8)	50 (1.8)	37 (2.0)	6 (1.3)	136 (1.8)
Liver disease	10 (1.5)	34 (1.8)	49 (1.8)	34 (1.8)	5 (1.1)	132 (1.7)
Malignant tumor	12 (1.8)	27 (1.5)	35 (1.3)	25 (1.4)	3 (0.7)	102 (1.3)
Respiratory disease	39 (5.9)	183 (9.8)	262 (9.5)	203 (11.0)	66 (14.5)	753 (9.9)
N/A	571 (85.6)	1534 (82.3)	2268 (82.3)	1513 (81.8)	371 (81.5)	6257 (82.4)
Atopic dermatitis, yes, *N* (%)	91 (13.6)	297 (15.9)	493 (17.9)	367 (19.9)	113 (24.8)	1361 (17.9)
Mental illness, *N* (%)						
No	620 (93.0)	1707 (91.6)	2456 (89.2)	1549 (83.8)	350 (76.9)	6682 (88.0)
Yes	14 (2.1)	69 (3.7)	117 (4.3)	163 (8.8)	63 (13.9)	426 (5.6)
Previously had	33 (5.0)	88 (4.7)	182 (6.6)	137 (7.4)	42 (9.2)	482 (6.4)
History of dry eye diagnosis, *N* (%)						
No	422 (63.3)	1037 (55.6)	1344 (48.8)	873 (47.2)	195 (42.9)	3871 (51.0)
Yes	121 (18.1)	394 (21.1)	678 (24.6)	473 (25.6)	128 (28.1)	1794 (23.6)
Unknown	124 (18.6)	433 (23.2)	733 (26.6)	503 (27.2)	132 (29.0)	1925 (25.4)

Abbreviations: Face score, the face scale score of the JACQLQ Domain III (0–4); N/A, not applicable; SD, standard deviation.

**TABLE 2:** Demographics and characteristics of participants by stress level category.

	Stress level scale 0–2	Stress level scale 3–4	Stress level scale 5–6	Stress level scale 7–8	Stress level scale 9–10	Overall
	*N* (%) = 2375 (31.3)	*N* (%) = 1239 (16.3)	*N* (%) = 1685 (22.2)	*N* (%) = 1702 (22.4)	*N* (%) = 589 (7.8)	*N* (%) = 7590 (100)
Demographic characteristics						
Age, years, mean (SD)	39.5 (13.7)	36.0 (14.3)	35.3 (13.5)	30.6 (12.6)	29.9 (12.2)	35.3 (13.9)
Women, *N* (%)	1176 (49.5)	699 (56.4)	941 (55.9)	1104 (64.9)	411 (69.8)	4331 (57.1)
Height, cm, mean (SD)	164.9 (8.8)	163.7 (8.7)	163.9 (8.8)	162.7 (8.8)	162.0 (8.7)	163.8 (8.8)
Weight, kg, mean (SD)	61.5 (12.2)	60.2 (12.0)	60.2 (12.0)	59.0 (12.3)	58.1 (12.9)	60.2 (12.3)
Medical history						
Medicated hypertension, *N* (%)						
No	1983 (83.4)	1033 (83.4)	1358 (80.6)	1400 (82.3)	451 (76.6)	6225 (82.0)
Medicated	164 (6.9)	76 (6.1)	86 (5.1)	47 (2.8)	13 (2.2)	386 (5.1)
Unmedicated	94 (4.0)	39 (3.2)	57 (3.4)	53 (3.1)	15 (2.6)	258 (3.4)
Unknown	134 (5.6)	91 (7.3)	184 (10.9)	202 (11.9)	110 (18.7)	721 (9.5)
Diabetes, *N* (%)						
No	2164 (91.1)	1131 (91.3)	1502 (89.1)	1508 (88.6)	514 (87.3)	6819 (89.9)
Yes	69 (2.9)	22 (1.8)	33 (2.0)	28 (1.7)	6 (1.0)	158 (2.1)
Unknown	142 (6.0)	86 (6.9)	150 (8.9)	166 (9.8)	69 (11.7)	613 (8.1)
Systemic diseases, yes, *N* (%)						
Blood disease	56 (2.4)	16 (1.3)	19 (1.1)	19 (1.1)	9 (1.5)	119 (1.6)
Brain disease	23 (1.0)	9 (0.7)	29 (1.7)	11 (0.7)	8 (1.4)	80 (1.1)
Collagen disease	10 (0.4)	10 (0.8)	9 (0.5)	8 (0.5)	4 (0.7)	41 (0.5)
Heart disease	54 (2.3)	26 (2.1)	27 (1.6)	32 (1.9)	16 (2.7)	155 (2.0)
Kidney disease	48 (2.0)	31 (2.5)	23 (1.4)	25 (1.5)	9 (1.5)	136 (1.8)
Liver disease	45 (1.9)	30 (2.4)	25 (1.5)	24 (1.4)	8 (1.4)	132 (1.7)
Malignant tumor	43 (1.8)	16 (1.3)	21 (1.3)	18 (1.1)	4 (0.7)	102 (1.3)
Respiratory disease	228 (9.6)	135 (10.9)	160 (9.5)	157 (9.2)	73 (12.4)	753 (9.9)
N/A	1931 (81.3)	998 (80.6)	1410 (83.7)	1438 (84.5)	480 (81.5)	6257 (82.4)
Atopic dermatitis, yes, *N* (%)	375 (15.8)	207 (16.7)	320 (19.0)	332 (19.5)	127 (21.6)	1361 (17.9)
Mental illness, *N* (%)						
No	2165 (91.2)	29 (2.3)	1474 (87.5)	1461 (85.8)	484 (82.2)	6682 (88.0)
Yes	87 (3.7)	1098 (88.6)	101 (6.0)	121 (7.1)	62 (10.5)	426 (5.6)
Previously had	123 (5.2)	55 (4.4)	110 (6.5)	120 (7.1)	43 (7.3)	482 (6.4)
History of dry eye diagnosis, *N* (%)						
No	1363 (57.4)	86 (6.9)	858 (50.9)	784 (46.1)	250 (42.4)	3871 (51.0)
Yes	499 (21.0)	616 (49.7)	405 (24.0)	427 (25.1)	158 (26.8)	1794 (23.6)
Unknown	513 (21.6)	305 (24.6)	422 (25.0)	491 (28.9)	181 (30.7)	1925 (25.4)

Abbreviations: N/A, not applicable; SD, standard deviation.

### 3.2 Subjective symptom scores

A summary of subjective symptom scores is shown in Tables 3 and 4. The mean scores (±SD) of the NSS, NNSS, and TSS were 4.7 (±4.0), 2.4 (±2.6), and 7.1 (±6.0), respectively. The means of symptom scores in all items were higher in the group with more severe face scores and higher stress levels. Most of the included hay‐fever‐related data were entered in February or March of each year (Supplementary Figure 3), which is the annual season with the most increased dispersion of cedar pollen and cypress pollen in Japan.^4^, ^44^

**TABLE 3:** Subjective symptom scores of participants by face scale score category.

Symptoms	Face score 0	Face score 1	Face score 2	Face score 3	Face score 4	Overall
	*N* (%) = 667 (8.8)	*N* (%) = 1864 (24.6)	*N* (%) = 2755 (36.3)	*N* (%) = 1849 (24.4)	*N* (%) = 455 (6.0)	*N* (%) = 7590 (100)
Nasal symptom score, 0–3, mean (SD)						
Rhinorrhea	0.4 (0.6)	0.8 (0.8)	1.1 (0.9)	1.5 (1.0)	1.8 (1.1)	1.1 (1.0)
Nasal congestion	0.3 (0.6)	0.6 (0.8)	0.9 (0.9)	1.3 (1.0)	1.6 (1.1)	0.9 (1.0)
Nasal itching	0.2 (0.5)	0.5 (0.8)	0.8 (0.9)	1.1 (1.0)	1.4 (1.1)	0.8 (0.9)
Sneezing	0.3 (0.6)	0.6 (0.8)	0.9 (0.9)	1.2 (1.0)	1.4 (1.1)	0.9 (1.0)
Effects on daily life	0.2 (0.6)	0.6 (0.8)	1.0 (0.9)	1.5 (1.0)	1.9 (1.2)	1.0 (1.0)
Total nasal symptom score, 0–15	1.2 (1.9)	3.2 (3.2)	4.7 (3.6)	6.6 (4.2)	8.1 (4.7)	4.7 (4.0)
Non‐nasal symptom score, 0–3, mean (SD)						
Eye itching	0.3 (0.6)	0.6 (0.8)	1.0 (1.0)	1.3 (1.1)	1.5 (1.1)	0.9 (1.0)
Tearing	0.1 (0.4)	0.3 (0.6)	0.5 (0.7)	0.8 (0.9)	1.1 (1.1)	0.5 (0.8)
Eye redness	0.1 (0.3)	0.3 (0.6)	0.6 (0.7)	0.8 (0.9)	1.0 (1.0)	0.6 (0.8)
Ear and/or mouse itching	0.1 (0.4)	0.2 (0.6)	0.4 (0.7)	0.6 (0.9)	0.8 (1.0)	0.4 (0.8)
Total non‐nasal symptoms score, 0–12	0.6 (1.2)	1.5 (2.0)	2.4 (2.4)	3.5 (2.9)	4.4 (3.4)	2.4 (2.6)
Total nasal and non‐nasal symptoms score, 0–27	2.0 (3.1)	4.7 (4.6)	7.1 (5.3)	10.1 (6.2)	12.5 (7.3)	7.1 (6.0)

Abbreviations: Face score, the face scale score of the JACQLQ Domain III (0–4); SD, standard deviation.

**TABLE 4:** Subjective symptom scores of participants by stress level category.

Symptoms	Stress level scale 0–2	Stress level scale 3–4	Stress level scale 5–6	Stress level scale 7–8	Stress level scale 9–10	Overall
	*N* (%) = 2375 (31.3)	*N* (%) = 1239 (16.3)	*N* (%) = 1685 (22.2)	*N* (%) = 1702 (22.4)	*N* (%) = 589 (7.8)	*N* (%) = 7590 (100)
Nasal symptom score, 0–3, mean (SD)						
Rhinorrhea	0.5 (0.6)	0.9 (0.7)	1.0 (0.9)	1.7 (1.0)	2.2 (1.0)	1.1 (1.0)
Nasal congestion	0.4 (0.6)	0.7 (0.8)	0.9 (0.9)	1.4 (1.0)	1.9 (1.1)	0.9 (1.0)
Nasal itching	0.3 (0.6)	0.7 (0.7)	0.7 (0.8)	1.3 (1.0)	1.7 (1.2)	0.8 (0.9)
Sneezing	0.4 (0.6)	0.8 (0.7)	0.8 (0.9)	1.4 (1.0)	1.9 (1.1)	0.9 (1.0)
Effects on daily life	0.3 (0.6)	0.8 (0.7)	0.9 (0.9)	1.7 (0.9)	2.3 (1.0)	1.0 (1.0)
Total nasal symptom score, 0–15	1.9 (2.1)	3.9 (2.6)	4.3 (3.3)	7.6 (3.8)	10.0 (4.3)	4.7 (4.0)
Non‐nasal symptom score, 0–3, mean (SD)						
Eye itching	0.4 (0.6)	0.8 (0.8)	0.9 (0.0)	1.1 (0.0)	1.1 (0.0)	0.9 (1.0)
Tearing	0.2 (0.4)	0.4 (0.6)	0.5 (0.7)	0.9 (0.9)	1.3 (1.1)	0.5 (0.8)
Eye redness	0.2 (0.5)	0.5 (0.6)	0.5 (0.7)	0.9 (0.8)	1.3 (1.1)	0.6 (0.8)
Ear and/or mouse itching	0.1 (0.3)	0.3 (0.6)	0.4 (0.7)	0.8 (0.9)	1.1 (1.1)	0.4 (0.8)
Total non‐nasal symptoms score, 0–12	0.9 (1.3)	2.0 (1.8)	2.3 (2.3)	4.0 (2.8)	5.4 (3.5)	2.4 (2.6)
Total nasal and non‐nasal symptoms score, 0–27	2.8 (3.0)	5.9 (3.7)	6.7 (4.9)	11.6 (5.6)	15.5 (6.5)	7.1 (6.0)

Abbreviation: SD, standard deviation.

### 3.3 Estimation of the MCID with the anchor‐based method

The Spearman's correlation coefficients between anchor groups and NSS, NNSS, and TSS were higher than 0.3 for anchors of face scale score and stress level scale (in face scale score category: NSS 0.435, NNSS 0.384, TSS 0.463; in stress level category: NSS 0.596; NNSS 0.509, TSS 0.627). A total of 24 MCIDs, eight each in NSS, NNSS, and TSS, were calculated by the anchor‐based method (range: NSS 0.4–3.3, NNSS 0.3–1.7, TSS 0.8–4.9). MCIDs based on the anchor‐based method were summarized using median with IQR: NSS (2.0, 1.5–2.1), NNSS (1.0, 0.9–1.2), and TSS (2.9, 2.4–3.3). The summary of MCID estimates is shown in Table 5.

**TABLE 5:** Summary of MCID estimates.

	NSS	NNSS	TSS
Anchor‐based, mean difference			
Face scale score 1–0	2.0	0.9	2.7
Face scale score 2–1	1.5	0.9	2.4
Face scale score 3–2	1.9	1.1	3.0
Face scale score 4–3	1.5	0.9	2.4
Stress level scale 3–4—0–2	2.0	1.1	3.1
Stress level scale 5–6—3–4	0.4	0.3	0.8
Stress level scale 7–8—5–6	3.3	1.7	4.9
Stress level scale 9–10—7–8	2.4	1.4	3.9
Median anchor‐based estimates (IQR)	2.0 (1.5–2.1)	1.0 (0.9–1.2)	2.9 (2.4–3.3)
Distribution‐based			
0.5‐SD	2.0	1.3	3.0
Standard error of measurement	1.8	1.2	2.3
MCID range	1.8–2.1	1.2–1.3	2.4–3.3

Abbreviations: IQR, interquartile range; MCID, minimal clinically important difference; NNSS, non‐nasal symptom score; NSS, nasal symptom score; SD, standard deviation; TSS, total symptom score.

### 3.4 Estimation of the MCID with two distribution‐based methods

The results of the distribution‐based analysis are shown in Table 5. MCID estimates based on half an SD at baseline were 2.0, 1.3, and 3.0 for NSS, NNSS, and TSS, respectively. ICC values were analyzed to determine the SEM from the data of a total of 286 participants (mean age ± SD [38.8 ± 14.7], and 50.0% of the participants were women). ICCs were 0.808 (95% confidence interval [95% CI], 0.763–0.846), 0.795 (95% CI, 0.748–0.834), and 0.849 (0.814–0.879) for NSS, NNSS, and TSS, respectively (Table 6). Based on the ICCs, the SEM was found to be 1.8 for NSS, 1.2 for NNSS, and 2.3 for TSS (Table 5).

**TABLE 6:** Test‐retest reliability of subjective symptom scores.

Questionnaires	Number of items	Day 1	Day 2	ICC (95% CI)
		Mean (SD)	Mean (SD)	
Nasal symptom score, 0–15	5	4.4 (4.0)	3.9 (3.8)	0.808 (0.763–0.846)
Non‐nasal symptom score, 0–12	4	2.3 (2.6)	2.4 (2.6)	0.795 (0.748–0.834)
Total nasal and non‐nasal symptoms score, 0–27	9	6.8 (6.0)	6.4 (5.9)	0.849 (0.814–0.879)

Abbreviations: CI, confidence interval; SD, standard deviation; ICC, intraclass correlation coefficient.

### 3.5 Estimation of the MCID ranges

The estimated SEM of 1.8 for NSS was larger than the 25th percentile of median anchor‐based estimates of 1.5, and the SEM was selected as the lower boundary of the MCID range for NSS. The estimated SEM of 1.2 for NNSS was equal to the 75th percentile of median anchor‐based estimates, and the MCIDs of NNSS calculated by the anchor‐based method were replaced by the SEM. The final suggested MCID ranges for NSS, NNSS, and TSS were 1.8–2.1, 1.2–1.3, and 2.4–3.3, respectively (Table 5).

## 4 DISCUSSION

To accurately assess ePROs and their clinical utility when collected through mHealth, MCIDs must be determined for each outcome in the context of mHealth. This study generated MCID ranges for NSS, NNSS, and TSS appropriate for mHealth‐based data. Using the suggested MCID values, users' hay‐fever symptoms may be more accurately monitored to evaluate treatment effects and facilitate regimen adjustments for each individual to maximize treatment efficacy.

This study generated a suggested MCID range of 1.8–2.1, 1.2–1.3, and 2.4–3.3 points for the 4‐point scale NSS, NNSS, and TSS, respectively. A previous study on the reported rhinoconjunctivitis total symptom score, which consists of a 6‐item questionnaire with each item rated on a 4‐point scale, suggested an MCID range of 1.1–1.3 (approximately 0.18 points per item).^13^ Another study on the MCID of NSS consisting of three 4‐point scale items suggested an MCID of 0.55 points (0.18 points per item).^22^ Compared to previous studies, the per‐item MCID for the collected app‐based TSS was larger (0.27–0.37 points per item).^11^ However, study designs that target patients visiting medical facilities may experience a higher degree of sampling homogeneity than the average population, and distribution‐based MCID ranges, which are highly influenced by the variability of collected PROs, may be narrower in such research designs.^45^ This study utilized a highly inclusive sampling method with no exclusion criteria based on hay‐fever severity, age, and location owing to the advantages of app‐based recruitment. Of the population providing data for this study, 83.6% (6344/7590) reported hay‐fever symptoms. Therefore, the resultant distribution‐based MCID is thought to better reflect the physiological undulations of hay‐fever symptoms experienced by the population, which consists primarily of symptomatic patients, and these MCID ranges may be more appropriate in assessing hay‐fever PROs for the general public than previously reported values. It is crucial to utilize MCID values that best reflect the targeting population, and hence, we suggested an MCID range rather than a single value for each PRO.^39^ In future studies that require assessment of meaningful changes to clinical status in hay‐fever patients, the suggested MCID range allows clinicians to select an MCID value suitable for the setting (for example, the lowest MCID value for specialty clinics) and accurately monitor hay‐fever progression.

In this study, two methods were utilized to calculate MCID: (1) distribution‐based method and (2) anchor‐based method. Among the two approaches, results from the anchor‐based method, which utilizes an external clinical marker termed the “anchor”, are prioritized in current practices.^22^, ^24^ This is in part due to the unwanted influence of various characteristics of the subject pool when relying solely on the distribution‐based method. However, anchor‐based methods are prone to subjectivity from researchers during “anchor” selection,^22^ and the distribution‐based approach is relatively bias‐free and free from the subjectivity of researchers.^22^, ^24^ Therefore, combining the two strategies to determine MCID values is preferred.^22^, ^24^ For an anchor‐based method, it is recommended to assign multiple anchors for outcomes, and for a distribution‐based method, calculating MCID using SD and SEM may yield a more accurate MCID that reflects the constant variability of PROs.^24^ Additionally, a larger sample size may help estimate a more accurate MCID range.^25^ In this study, we selected two anchors—face score of JACQLQ domain III and stress score—for the anchor‐based method and utilized both the SD and SEM for the distribution‐based method, approaching MCID calculation in a multi‐faceted manner. The smartphone app‐based, large‐scale design is also a strength of this study and the resultant MCID ranges.^4^ Using the suggested MCID values from this study, clinicians and researchers may be able to analyze hay‐fever symptom reports to determine treatment effects more meaningfully and accurately through the mHealth app.

This study had several limitations. First, the designated anchors were selected based on results from a cross‐sectional study and may not be strongly correlated with the fluctuating hay‐fever symptoms assessed in a longitudinal fashion. Future studies should validate the accuracy of the suggested MCID values with changing symptoms when followed long‐term.^39^ Second, every clinical marker used to assess hay‐fever status in this study was a PRO, and data on objective physical findings and clear diagnosis of hay fever were not used. Therefore, future studies should incorporate relevant clinical findings and clear diagnoses from physical examinations to calculate MCID values and compare this with this study's results to prevent errors due to subjective patient assessment.^23^ Third, the MCID for this study was calculated from data collected using AllerSearch from a Japanese population with mild to moderate (TSS: 7.1 ± 6.1) subjective symptoms of hay fever. The results of ePRO can vary if the layout of the questionnaire differs significantly, even if the questions remain the same.^46^ Therefore, the MCID calculated in this study may not be applicable, for example, to non‐Japanese patient populations with severe hay‐fever symptoms and using other applications that have a significantly different questionnaire layout than that of AllerSearch. On the other hand, the MCID in this study may be applicable to a Japanese population with mild to moderate hay‐fever symptoms using an ePRO application that has a questionnaire with a layout similar to that of the NSS and NNSS questionnaires in AllerSearch. Fourth, this study used only data collected from the iOS version of AllerSearch. The Android version of AllerSearch has been available since August 2020, and additional data from the Android version of AllerSearch may increase the sample size and improve the results. However, as the sample size for this study is large, the MCID in this study may also be appropriate. In addition, participants with more severe symptoms in this study tended to report an unknown medical history. This may indicate that symptom severity is occurring in unreached patients who are not receiving appropriate diagnosis and treatment for hay fever.^32^ The combination of MCID and mHealth apps has the potential to provide appropriate hay‐fever treatment with remote subjective symptom assessment to previously unreached patients. Fifth, hay‐fever severity may vary depending on when the information was registered, but this study also includes data from various other seasons. However, most of included hay‐fever symptom data were registered in February and March. Therefore, the MCID in this study may be applicable to cedar and cypress pollen‐allergic patients, which constitute the majority of the Japanese hay‐fever patients.

In conclusion, our study results suggest MCID ranges of 1.8–2.1, 1.2–1.3, and 2.4–3.3 points for NSS, NNSS, and TSS, respectively. The MCID ranges from this study were based on data from a smartphone app‐based, large‐scale clinical research in the Japanese population, and were calculated utilizing a multi‐faceted approach. This may have implications as a useful standard in the global shift toward incorporating mHealth when evaluating hay‐fever symptoms and monitoring treatment effectiveness in practice and research.

## AUTHOR CONTRIBUTIONS

**Ken Nagino**: Conceptualization (lead); data curation (equal); formal analysis (lead); methodology (lead); software (lead); validation (lead); visualization (lead); writing—original draft preparation (lead). **Jaemyoung Sung**: writing—original draft preparation (equal); writing—review & editing (equal). **Akie Midorikawa‐Inomata**: funding acquisition (equal); resources (equal); writing—review & editing (equal). **Atsuko Eguchi**: funding acquisition (equal); resources (equal); writing—review & editing (equal). **Keiichi Fujimoto**: funding acquisition (equal); resources (equal); writing—review & editing (equal). **Yuichi Okumura**: project administration (equal); writing—review & editing (equal). **Alan Yee**: original draft preparation (equal); writing—review & editing (equal). **Kenta Fujio**: data curation (equal); writing—review & editing (equal). **Yasutsugu Akasaki**: data curation (equal); writing—review & editing (equal). **Tianxiang Huang**: data curation (equal); writing—review & editing (equal). **Maria Miura**: data curation (equal); writing—review & editing (equal). **Shokirova Hurramhon**: funding acquisition (equal); resources (equal); writing—review & editing (equal). **Kunihiko Hirosawa**: data curation (equal); writing—review & editing (equal). **Mizu Ohno**, data curation (equal); writing—review & editing (equal). **Yuki Morooka**, data curation (equal); writing—review & editing (equal). **Hiroyuki Kobayashi**: project administration (equal); resources (equal); supervision (equal). **Takenori Inomata**: Conceptualization (equal); funding acquisition (lead); investigation (lead); project administration (lead); resources (lead); supervision (lead); writing—review & editing (lead).

## CONFLICT OF INTEREST STATEMENT

The AllerSearch application was created using Apple's ResearchKit (Cupertino, CA, USA). Takenori Inomata, Yuichi Okumura, and Akie Midorikawa‐Inomata are the owners of InnoJin, Inc., Tokyo, Japan, which developed AllerSearch. Takenori Inomata report receiving grants from Johnson & Johnson Vision Care, SEED Co., Ltd., Novartis Pharma K.K., and Kowa Company, Ltd., outside the submitted work, as well as personal fees from Santen Pharmaceutical Co., Ltd., and InnoJin, Inc. Ken Nagino, Yuichi Okumura., and Akie Midorikawa‐Inomata received personal fees from InnoJin, Inc. outside the submitted work. The remaining authors declare no competing interests.

## Supporting information

Supplementary Material

## References

[1] Okuda M . Epidemiology of Japanese cedar pollinosis throughout Japan. Ann Allergy Asthma Immunol. 2003;91(3):288‐296. 10.1016/s1081-1206(10)63532-6 14533662

[2] Sakashita M , Hirota T , Harada M , et al. Prevalence of allergic rhinitis and sensitization to common aeroallergens in a Japanese population. Int Arch Allergy Immunol. 2010;151(3):255‐261. 10.1159/000242363 19786806

[3] Mortimer K , Lesosky M , García‐Marcos L , et al. The burden of asthma, hay fever and eczema in adults in 17 countries: GAN phase I study. Eur Respir J. 2022;60(3):2102865. 10.1183/13993003.02865-2021 35210319 PMC9474894

[4] Inomata T , Nakamura M , Iwagami M , et al. Individual characteristics and associated factors of hay fever: a large‐scale mHealth study using AllerSearch. Allergol Int. 2022;71(3):325‐334. 10.1016/j.alit.2021.12.004 35105520

[5] von Mutius E . The environmental predictors of allergic disease. J Allergy Clin Immunol. 2000;105(1):9‐19. 10.1016/s0091-6749(00)90171-4 10629447

[6] Rosenkranz RR , Rosenkranz SK , Neessen KJ . Dietary factors associated with lifetime asthma or hayfever diagnosis in Australian middle‐aged and older adults: a cross‐sectional study. Nutr J. 2012;11(1):84. 10.1186/1475-2891-11-84 23057785 PMC3544658

[7] Upperman CR , Parker JD , Akinbami LJ , et al. Exposure to extreme heat events is associated with increased hay fever prevalence among nationally representative sample of US adults: 1997‐2013. J Allergy Clin Immunol Pract. 2017;5(2):435‐441.e432. 10.1016/j.jaip.2016.09.016 27840238 PMC5346329

[8] Ferreira MA , Matheson MC , Tang CS , et al. Genome‐wide association analysis identifies 11 risk variants associated with the asthma with hay fever phenotype. J Allergy Clin Immunol. 2014;133(6):1564‐1571. 10.1016/j.jaci.2013.10.030 24388013 PMC4280183

[9] Yamada T , Saito H , Fujieda S . Present state of Japanese cedar pollinosis: the national affliction. J Allergy Clin Immunol. 2014;133(3):632‐639.e635. 10.1016/j.jaci.2013.11.002 24361081

[10] Platt M . Pharmacotherapy for allergic rhinitis. Int Forum Allergy Rhinol. 2014;4(Suppl 2):S35‐S40. 10.1002/alr.21381 25182353

[11] Higaki T , Okano M , Kariya S , et al. Determining minimal clinically important differences in Japanese cedar/cypress pollinosis patients. Allergol Int. 2013;62(4):487‐493. 10.2332/allergolint.13-oa-0570 24153331

[12] Akasaki Y , Inomata T , Sung J , et al. Reliability and validity of electronic patient‐reported outcomes using the smartphone app AllerSearch for hay fever: prospective observational study. JMIR Form Res. 2022;6(8):e38475. 10.2196/38475 35998022 PMC9449823

[13] Devillier P , Chassany O , Vicaut E , et al. The minimally important difference in the Rhinoconjunctivitis Total Symptom Score in grass‐pollen‐induced allergic rhinoconjunctivitis. Allergy. 2014;69(12):1689‐1695. 10.1111/all.12518 25155425

[14] U.S. Department of Health and Human Services FDA Center for Drug Evaluation and Research . Guidance for industry: patient‐reported outcome measures: use in medical product development to support labeling claims: draft guidance. Health Qual Life Outcomes. 2006;4(1):79. 10.1186/1477-7525-4-79 17034633 PMC1629006

[15] Kirtsreesakul V , Somjareonwattana P , Ruttanaphol S . The correlation between nasal symptom and mucociliary clearance in allergic rhinitis. Laryngoscope. 2009;119(8):1458‐1462. 10.1002/lary.20146 19507239

[16] Juniper EF , Thompson AK , Ferrie PJ , Roberts JN . Validation of the standardized version of the rhinoconjunctivitis quality of life questionnaire. J Allergy Clin Immunol. 1999;104(2):364‐369. 10.1016/s0091-6749(99)70380-5 10452758

[17] Fukagawa K . How to use Japanese allergic conjunctival disease quality‐of‐life questionnaire (JACQLQ). Arerugi. 2014;63:764‐766. 24953735

[18] Bottomley A , Jones D , Claassens L . Patient‐reported outcomes: assessment and current perspectives of the guidelines of the Food and Drug Administration and the reflection paper of the European Medicines Agency. Eur J Cancer. 2009;45(3):347‐353. 10.1016/j.ejca.2008.09.032 19013787

[19] Okumura Y , Inomata T , Iwata N , et al. A review of dry eye questionnaires: measuring patient‐reported outcomes and health‐related quality of life. Diagnostics (Basel). 2020;10(8):559. 10.3390/diagnostics10080559 32764273 PMC7459853

[20] Inomata T , Nakamura M , Iwagami M , et al. Symptom‐based stratification for hay fever: a crowdsourced study using the smartphone application AllerSearch. Allergy. 2021;76(12):3820‐3824. 10.1111/all.15078 34480802

[21] Jaeschke R , Singer J , Guyatt GH . Measurement of health status. Ascertaining the minimal clinically important difference. Control Clin Trials. 1989;10(4):407‐415. 10.1016/0197-2456(89)90005-6 2691207

[22] Barnes ML , Vaidyanathan S , Williamson PA , Lipworth BJ . The minimal clinically important difference in allergic rhinitis. Clin Exp Allergy. 2010;40(2):242‐250. 10.1111/j.1365-2222.2009.03381.x 19895590

[23] Kerezoudis P , Yost KJ , Tombers NM , Celda MP , Carlson ML , Link MJ . Defining the minimal clinically important difference for patients with vestibular Schwannoma: are all quality‐of‐life scores significant? Neurosurgery. 2019;85(6):779‐785. 10.1093/neuros/nyy467 30395303

[24] Sedaghat AR . Understanding the minimal clinically important difference (MCID) of patient‐reported outcome measures. Otolaryngol Head Neck Surg. 2019;161(4):551‐560. 10.1177/0194599819852604 31159641

[25] Wang YC , Hart DL , Stratford PW , Mioduski JE . Baseline dependency of minimal clinically important improvement. Phys Ther. 2011;91(5):675‐688. 10.2522/ptj.20100229 21372203

[26] Fujio K , Inomata T , Fujisawa K , et al. Patient and public involvement in mobile health‐based research for hay fever: a qualitative study of patient and public involvement implementation process. Res Involv Engage. 2022;8(1):45. 10.1186/s40900-022-00382-6 PMC9437402 36056430

[27] Okumura Y , Inomata T , Midorikawa‐Inomata A , et al. DryEyeRhythm: a reliable and valid smartphone application for the diagnosis assistance of dry eye. Ocul Surf. 2022;25:19‐25. 10.1016/j.jtos.2022.04.005 35483601

[28] Inomata T , Nakamura M , Sung J , et al. Smartphone‐based digital phenotyping for dry eye toward P4 medicine: a crowdsourced cross‐sectional study. NPJ Digit Med. 2021;4(1):171. 10.1038/s41746-021-00540-2 34931013 PMC8688467

[29] Inomata T , Nakamura M , Iwagami M , et al. Stratification of individual symptoms of contact lens‐associated dry eye using the iPhone app DryEyeRhythm: crowdsourced cross‐sectional study. J Med Internet Res. 2020;22(6):e18996. 10.2196/18996 32589162 PMC7381048

[30] Eguchi A , Inomata T , Nakamura M , et al. Heterogeneity of eye drop use among symptomatic dry eye individuals in Japan: large‐scale crowdsourced research using DryEyeRhythm application. Jpn J Ophthalmol. 2021;65(2):271‐281. 10.1007/s10384-020-00798-1 33411099

[31] Inomata T , Iwagami M , Nakamura M , et al. Association between dry eye and depressive symptoms: large‐scale crowdsourced research using the DryEyeRhythm iPhone application. Ocul Surf. 2020;18(2):312‐319. 10.1016/j.jtos.2020.02.007 32113987

[32] Inomata T , Iwagami M , Nakamura M , et al. Characteristics and risk factors associated with diagnosed and undiagnosed symptomatic dry eye using a smartphone application. JAMA Ophthalmol. 2020;138(1):58‐68. 10.1001/jamaophthalmol.2019.4815 31774457 PMC6902113

[33] Inomata T , Nakamura M , Iwagami M , et al. Risk factors for severe dry eye disease: crowdsourced research using DryEyeRhythm. Ophthalmology. 2019;126(5):766‐768. 10.1016/j.ophtha.2018.12.013 30550734

[34] Inomata T , Sung J , Nakamura M , et al. New medical big data for P4 medicine on allergic conjunctivitis. Allergol Int. 2020;69(4):510‐518. 10.1016/j.alit.2020.06.001 32651122

[35] Inomata T , Sung J , Fujio K , et al. Individual multidisciplinary clinical phenotypes of nasal and ocular symptoms in hay fever: Crowdsourced cross‐sectional study using AllerSearch. Allergol Int. 2023. 10.1016/j.alit.2023.01.001 36740498

[36] Ousmen A , Touraine C , Deliu N , et al. Distribution‐ and anchor‐based methods to determine the minimally important difference on patient‐reported outcome questionnaires in oncology: a structured review. Health Qual Life Outcome. 2018;16(1):228. 10.1186/s12955-018-1055-z PMC6288886 30537955

[37] Revicki D , Hays RD , Cella D , Sloan J . Recommended methods for determining responsiveness and minimally important differences for patient‐reported outcomes. J Clin Epidemiol. 2008;61(2):102‐109. 10.1016/j.jclinepi.2007.03.012 18177782

[38] Farrar JT , Young JP, Jr. , LaMoreaux L , Werth JL , Poole MR . Clinical importance of changes in chronic pain intensity measured on an 11‐point numerical pain rating scale. Pain. 2001;94(2):149‐158. 10.1016/s0304-3959(01)00349-9 11690728

[39] Carlson ML , Tveiten ØV , Yost KJ , Lohse CM , Lund‐Johansen M , Link MJ . The minimal clinically important difference in vestibular Schwannoma quality‐of‐life assessment: an important step beyond P < .05. Otolaryngol Head Neck Surg. 2015;153(2):202‐208. 10.1177/0194599815585508 26038393

[40] Stark RG , Reitmeir P , Leidl R , König H.‐H . Validity, reliability, and responsiveness of the EQ‐5D in inflammatory bowel disease in Germany. Inflamm Bowel Dis. 2009;16(1):42‐51. 10.1002/ibd.20989 19475674

[41] Norman GR , Sloan JA , Wyrwich KW . Interpretation of changes in health‐related quality of life: the remarkable universality of half a standard deviation. Med Care. 2003;41(5):582‐592. 10.1097/01.mlr.0000062554.74615.4c 12719681

[42] Asher AL , Kerezoudis P , Mummaneni PV , et al. Defining the minimum clinically important difference for grade I degenerative lumbar spondylolisthesis: insights from the Quality Outcomes Database. Neurosurg Focus. 2018;44(1):E2. 10.3171/2017.10.focus17554 29290132

[43] Sagberg LM , Jakola AS , Solheim O . Quality of life assessed with EQ‐5D in patients undergoing glioma surgery: what is the responsiveness and minimal clinically important difference? Qual Life Res. 2014;23(5):1427‐1434. 10.1007/s11136-013-0593-4 24318084

[44] Okamoto Y , Horiguchi S , Yamamoto H , Yonekura S , Hanazawa T . Present situation of cedar pollinosis in Japan and its immune responses. Allergol Int. 2009;58(2):155‐162. 10.2332/allergolint.08-rai-0074 19307773

[45] van Veelen A , Abtahi S , Souverein P , et al. Characteristics of patients with lung cancer in clinical practice and their potential eligibility for clinical trials evaluating tyrosine kinase inhibitors or immune checkpoint inhibitors. Cancer Epidemiol. 2022;78:102149. 10.1016/j.canep.2022.102149 35429893

[46] Coons SJ , Gwaltney CJ , Hays RD , et al. Recommendations on evidence needed to support measurement equivalence between electronic and paper‐based patient‐reported outcome (PRO) measures: ISPOR ePRO Good Research Practices Task Force report. Value Health. 2009;12(4):419‐429. 10.1111/j.1524-4733.2008.00470.x 19900250

