# Supplementary material for: The minimal clinically important difference of app‐based electronic patient‐reported outcomes for hay fever
Source: Clin Transl Allergy. 2023 May 1;13(5):e12244. doi: 10.1002/clt2.12244 (PMC10151605; doi:10.1002/clt2.12244)
Supplement: Supplementary file 1 — Supplementary Material [file CLT2-13-e12244-s001.docx]

## Supplementary Data

**SUPPLEMENTARY FIGURES**


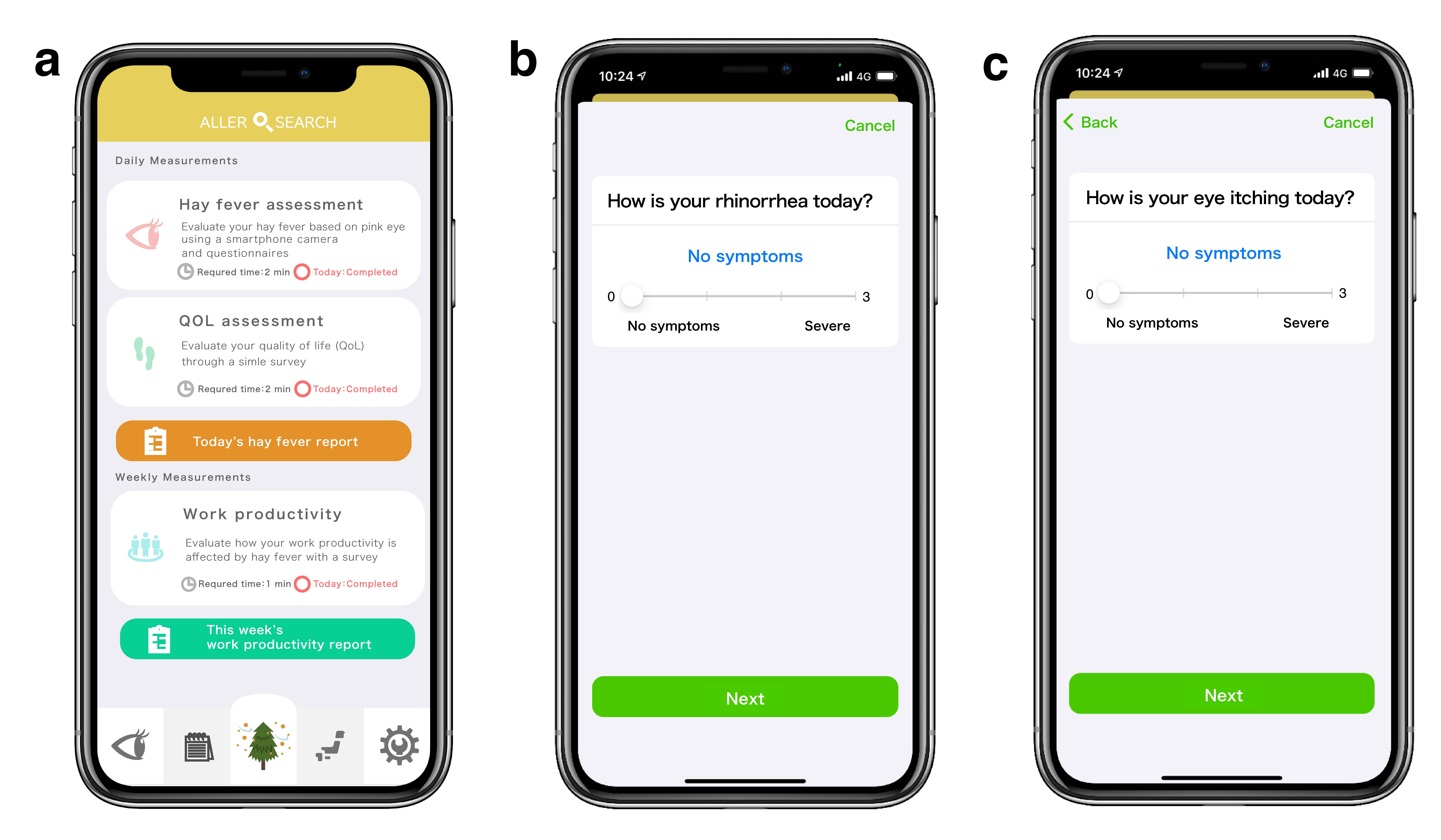
**Supplementary Figure. 1.** **Screenshots of the AllerSearch application.** Screenshots of the (a) top screen, (b) nasal symptoms score, and (c) non-nasal symptoms score.

**Supplementary Figure. 2. Schematic representation of participant enrollment, exclusion, and inclusion in this study.**

**Supplementary Figure. 3. Number of data entries for the first time in the study period.**

**SUPPLEMENTARY TABLES**

**Supplementary Table 1. Survey questions**

| **Questions** | **Variables** | **Details of variables** |
| --- | --- | --- |
| **User characteristics** |  |  |
|  | Age | Integer input, years |
|  | Sex | Choose one {“Man,” “Woman”} |
|  | Height | Integer input, cm |
|  | Weight | Integer input, kg |
| **Medical history** |  |  |
| Have you ever been diagnosed with hypertension? | Medicated hypertension | Choose one {“No,” “I am being treated for hypertension,” “I have untreated hypertension,” “I do not know”} |
| Have you ever been diagnosed with diabetes? | Diabetes (HbA1c level) | Choose one {“Yes,” “No,” “I do not know”}. If “Yes,” scale bar input of HbA1c level (5–15%) |
| Have you experienced any of the following illnesses? | Systemic diseases | Multiple choice among {“Heart disease,” “Respiratory disease,” “Brain disease,” “Liver disease,” “Kidney disease,” 'Blood disease,” “Malignant tumor,” “Collagen disease,” “N/A”} |
| If you have eczema (atopic dermatitis), when did it start? | Atopic dermatitis | Choose one {“Infancy (before 1 year of age),” “Early childhood (age 1–6 years),” “Middle childhood (age 7–12 years),” Early adolescence (after the age of 13 years)”} |
| Do you have any mental illness? | Mental illness | Choose one {“No,” “Yes”, “Previously had”} |
| Do you have any of the following mental illnesses? | Mental illness | If “Yes” in the previous question, multiple choice among {“Depression,” “Schizophrenia,” “Other mental illness”} |
| Have you ever been diagnosed with dry eye disease? | Dry eye disease | Choose one {“No,” “Yes,” “I do not know”} |
| **Hay fever** |  |  |
| Do you have hay fever? | Hay fever | Choose one {“Yes,” “No,” “Unknown”} |
| Please tell us which of the following hay fever prevention methods you have been using. | Preventive behavior | Multiple choice among {“Mask,” “Eye drops,” “Nasal spray/drops,” “Medication,” “Air purifier,” “Glasses and goggles,” “Other,” “Not using any”} |

**Abbreviations:** N/A, not applicable**.**

**Supplementary Table 2. Daily hay fever subjective symptom questionnaire**

| **Questions** | **Variables** | **Details of variables** |
| --- | --- | --- |
| **Nasal symptoms score** |  |  |
| Please rate how your rhinorrhea has been over the past 24 hours? | NSS item 1 | Choose one {“No symptoms,” “Mild symptoms (symptoms clearly present but easily tolerated,” “Moderate symptoms (symptoms bothersome but tolerable),” “Severe symptoms (symptoms difficult to tolerate—interfere with activities)”} |
| Please rate how your nasal congestion has been over the past 24 hours? | NSS item 2 |  |
| Please rate how your nasal itching has been over the past 24 hours? | NSS item 3 |  |
| Please rate how your sneezing has been over the past 24 hours? | NSS item 4 |  |
| How severely does hay fever affect your daily activities? | NSS item 5 |  |
| How much eye itching did you experience over the past 24 hours? | NNSS item 1 |  |
| How much eye-watering did you experience over the past 24 hours? | NNSS item 2 |  |
| How much eye redness did you experience over the past 24 hours? | NNSS item 3 |  |
| How much itching of the ear and nose did you experience over the past 24 hours? | NNSS item 4 |  |

**Abbreviations:** NSS, nasal symptom score; NNSS, non-nasal symptom score
